# Shear Deformation Behavior of a Double-Layer Asphalt Mixture Based on the Virtual Simulation of a Uniaxial Penetration Test

**DOI:** 10.3390/ma13173700

**Published:** 2020-08-21

**Authors:** Changjiang Kou, Xiaohui Pan, Peng Xiao, Aihong Kang, Zhengguang Wu

**Affiliations:** College of Civil Science and Engineering, Yangzhou University, Yangzhou 225100, China; changjiang.kou@yzu.edu.cn (C.K.); xiaohui.pan@163.com (X.P.); ahkang@yzu.edu.cn (A.K.); zgwu@yzu.edu.cn (Z.W.)

**Keywords:** double-layer asphalt mixture, shear deformation behavior, virtual uniaxial penetration test, discrete element model

## Abstract

This paper aims to clarify the shear deformation behavior of double-layer asphalt mixtures using the virtual uniaxial penetration test (UPT) with a discrete element method. For this purpose, asphalt mixtures with two different nominal maximum aggregate sizes were designed for the preparation of double-layer wheel tracking test specimens. Then, the cylindrical cores were prepared from the specimens and were cut for capturing the longitudinal profile images. These images were used to reconstruct a two-dimensional discrete element model (DEM) of the uniaxial penetration test specimen. The results indicate that the shear deformation behavior of the asphalt mixtures showed corresponding changes under the virtual loading. The tensile and compressive stress were distributed unevenly within the upper layer after the test, and both coarse aggregates and asphalt mortars bore a greater shear stress. Therefore, cracks were more likely to occur in the upper layer, leading to the failure of the specimens. This process enhanced the bonding between the asphalt mortars and the mineral aggregates. The aggregate particles in the upper layer moved more vertically, while those in the lower layer generally moved more laterally under the virtual loading. This behavior reveals the rutting mechanism of asphalt pavement.

## 1. Introduction

Rutting is known to represent a serious type of asphalt pavement distress that influences the safety of the road and the quality of the ride. In the SHRP-A-318 research report, pavement rutting is defined as the result of the combination of the compaction deformation and the transverse flow deformation of the asphalt mixture under numerous repeated traffic loadings [1,2]. B. Birgisson pointed out that the rutting was caused primarily by the shear deformation of the upper layer asphalt mixtures due to the failure to resist frequent repeated shear stress, which is now generally accepted by most road engineers [3].

In the past several decades, the triaxial compression test and rotary shear test were employed to analyze the shear performance of asphalt mixtures [4,5,6,7]. However, it is difficult to determine the exact shear strength of asphalt mixtures by shear performance analysis. The composition of an asphalt mixture, the internal structure formed by the joint action of the aggregates, and the service conditions are the internal factors that determine its bearing capacity and service performance. Traditional tests cannot evaluate the effect of asphalt binders on the shear strength, and cannot reflect the shear mechanism of the asphalt mixture through the damage process of the test sample. In comparison, the uniaxial penetration test (UPT) method proposed by Y.F. Bi and L.J. Sun can determine the shear strength of an asphalt mixture in the pavement structure, simulate the actual shear states of the road, and better reflect the shear strength of asphalt mixtures [8]. Chen et al. found that the uniaxial penetration test can provide repeatable results for the shear resistance of asphalt mixtures at elevated temperatures [9]. However, it is difficult to control the variability of experimental tests due to complex artificial and environmental factors, and to build relationships between the macro-properties and micro-structure of asphalt mixtures based on experimental tests. Micromechanical modeling and virtual testing are necessary to solve the above problems and to characterize the asphalt mixture for both material evaluation and structural design purposes.

Currently, the finite element method (FEM) and the discrete element method (DEM) are the two major numerical tools used to study the mechanical behavior of asphalt mixtures. Continuum theory is the basis of FEM models with random aggregate structures for asphalt mixtures. It is difficult to characterize the contact and sliding behavior between aggregates, and the bonding behavior between aggregates and asphalt mastic. The DEM method, initially developed by P.A. Cundall and O.L. Stack, provides a promising way to model and characterize the micromechanical behavior of asphalt mixtures [10]. 

DEM has been widely used to study the mechanical properties of granular materials and to solve the engineering problems of granular and discontinuous materials in recent years [11,12,13,14,15,16]. M. Enad used the discrete element method to analyze the impact of the aggregate gradation, shape, stiffness, and internal structure on asphalt mixture fractures [17]. S. Hou explained how asphalt mixtures bear vehicle loadings, as well as the potential reasons the rutting forms from a micro-mechanical view, through the micro-mechanical response of asphalt mixtures based on the discrete element method [18]. H. Feng studied the normal and shear material properties for a viscoelastic model of an asphalt mixture using DEM [19]. T. Ma et al. studied the effect of air voids on the high-temperature creep behavior and fatigue life of an asphalt mixture based on the discrete element method [20,21]. J.L. Ren and L.J. Sun used the discrete element method to characterize the effect of air voids on the cracking of asphalt concrete at low temperatures [22]. 

Until now, most investigations have focused on the micromechanical behavior of single-layer asphalt mixtures. In fact, an asphalt pavement surface usually consists of two or more layers. Therefore, a double-layer asphalt mixture can better reflect the deformation behavior similar to real pavement conditions. This study aims to analyze the shear deformation behavior of a double-layer asphalt mixture using the virtual uniaxial penetration test with a discrete element model. For this purpose, asphalt mixtures with two different nominal maximum aggregate sizes were designed for the preparation of the double-layer wheel tracking test specimens, which were then cored to obtain the cylindrical uniaxial penetration test specimens. The longitudinal profile images captured from cutting the cylindrical uniaxial penetration test specimens were used to reconstruct the two-dimensional virtual UPT specimens for the discrete element method analysis. In addition, the micromechanical properties and the spatial movement characteristics of the coarse aggregates in the specimens were analyzed to reveal new insights regarding the rutting failure mechanism of asphalt pavements.

## 2. Materials and Methods 

### 2.1. Materials

SBS modified asphalt with 80/100 penetration grade, basalt aggregates, limestone mineral powder, and flocculent lignin fibers were used to prepare a stone mastic asphalt mixture with a nominal maximum aggregate size of 13.2 mm (SMA13). The content of the lignin fibers was 0.3% of the total weight of the asphalt mixture. Hot mix asphalt with a nominal maximum aggregate size of 19.0 mm (AC20) was prepared using virgin asphalt with 60/70 penetration grade, limestone aggregates, and mineral powder. The sieving results of the aggregates are shown in Table 1 and Table 2.

**Figure 1:**
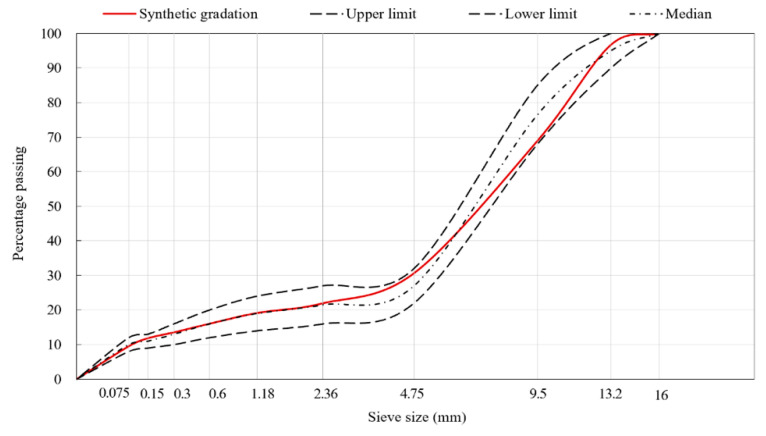
Aggregate grading curves of the SMA13 asphalt mixture.

**Figure 2:**
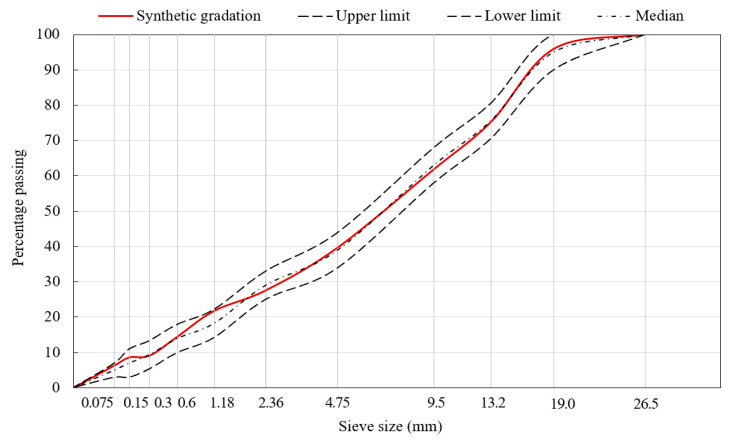
Aggregate grading curves of the AC20 asphalt mixture.

Following the Technical Specification for Construction of Highway Asphalt Pavement in China (JTG F40-2004) [23], the aggregate grading curves of SMA13 and AC20 were designed by controlling the critical sieve to 2.36 mm and 4.75 mm, respectively, within the effective ranges, as presented in Figure 1 and Figure 2, respectively. They were prepared based on the Marshall Mix Design method with a 4% targeted air void content. The asphalt content was 6.1% for SMA13 and 4.6% for AC20. The volumetric parameters and basic performances of SMA13 and AC20, as listed in Table 3, all met the standard requirements.

### 2.2. Specimen Preparation

**Figure 3:**
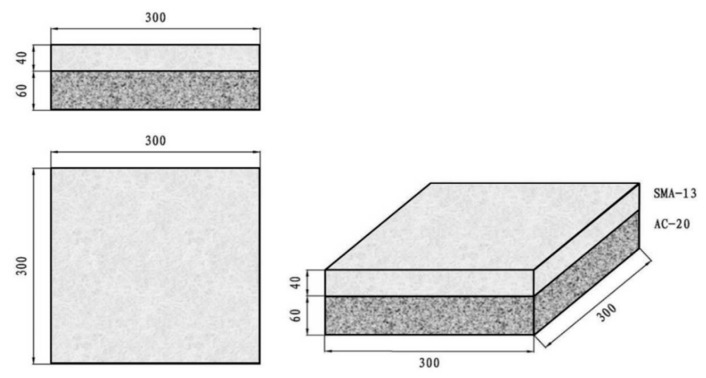
The size of the mold for the wheel tracking test specimen (mm).

Considering the size requirement of the wheel tracking tester and the height limitation of the surface layer combination, generally consisting of a 40 mm upper layer and a 60 mm lower layer, a mold, shown in Figure 3, with a 300 mm length, 300 mm width, and 100 mm height was designed to prepare the double-layer asphalt mixture. The upper layer was 40 mm SMA13 mixture and the lower layer was 60 mm AC20 mixture.

**Figure 4:**
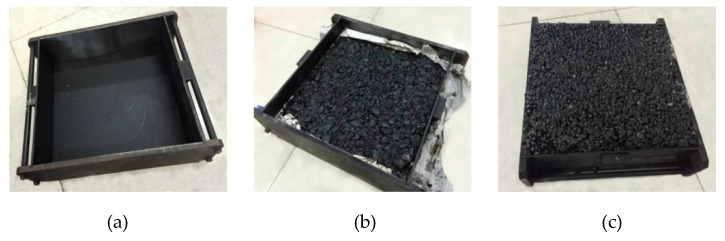
Preparation of the double-layer wheel tracking test specimen. (**a**) The mold for double-layer asphalt mixtures; (**b**) The compaction of the lower layer; (**c**) The compaction of a double-layer asphalt mixture.

Figure 4 presents the preparation process of the double-layer asphalt mixture specimen. Referring to the technical standard [24], for the lower layer, the temperature recommended for the mixing is between 140 ℃ to 160 ℃, and the compacting is between 120 ℃ to 150 ℃. For the upper layer, the temperature recommended for the mixing is between 160 ℃ to 175 ℃, and the compacting is between 140 ℃ to 170 ℃. In this study, the lower layer was mixed at 150 ℃, compacted at 140 ℃, and kept at room temperature for 24 h. The upper layer was mixed at 165 ℃ and compacted on the surface of the lower layer at 160 ℃. The whole specimen was kept at room temperature for 48 h.

**Figure 5:**
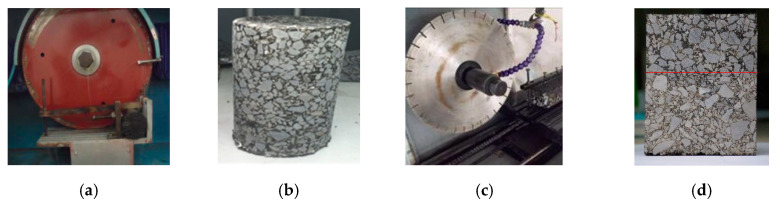
Longitudinal profile acquisition of the uniaxial penetration test specimen. (**a**) The coring machine; (**b**) The UPT specimen; (**c**) The cutting machine; (**d**) The longitudinal profile.

The double-layer wheel tracking test specimens were cored to obtain the cylindrical UPT specimens with a diameter of 100 mm, as shown in Figure 5. Figure 5 presents the longitudinal profile captured from cutting the cylindrical uniaxial penetration test specimen along the diameter direction, which was used to reconstruct the two-dimensional DEM of the virtual UPT specimen.

### 2.3. Laboratory Test

**Figure 6:**
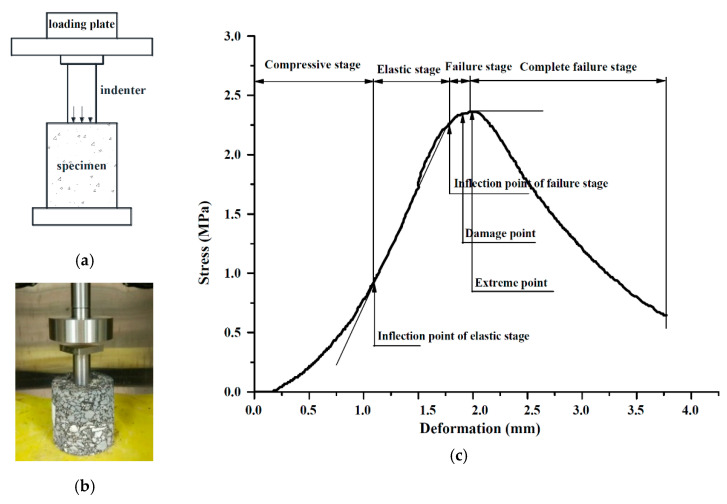
The loading mode, apparatus, and typical stress–deformation relationship of the UPT. (**a**) Loading mode of UPT; (**b**) An apparatus for UPT; (**c**) A typical stress–deformation relationship in the test.

The UPT method introduced by Y.F. Bi and L.J. Sun [8] was used in this research, and the maximum strength was defined as the uniaxial penetration strength of an asphalt mixture specimen. The UPT process, including the specimen size, penetration indenter size, and the transformation from penetration strength to shear strength, has been described in detail in the literature. Figure 6 shows the UPT, in which Figure 6a exhibits the loading mode of the UPT and Figure 6b shows the uniaxial penetration test apparatus. The indenter of the uniaxial penetration test was different from the ordinary shear test, and the upper part was a thin plate shape with a size of 5 cm × 5 cm × 1 cm. The lower part was a cylinder with dimensions of Φ 28.5 mm × 50 mm (for specimens with a diameter of 100 mm). Figure 6c illustrates the typical stress-deformation relationship in the UPT. The stress was calculated by dividing the ultimate load of the specimen at failure by the area, which is the cross-sectional area of the cylinder [25]. The test temperature for the standard uniaxial penetration test was 60 ℃. A Universal Testing Machine (UTM; IPC global, Victoria, Australia) was used to conduct a UPT at 60 ℃ to measure the maximum strength, which was regarded as the failure strength of an asphalt mixture specimen. The loading rate during the test was 1 mm/min [26].

## 3. Modeling of the Virtual Uniaxial Penetration Test

### 3.1. Capture and Processing of the Longitudinal Profile Images

**Figure 7:**
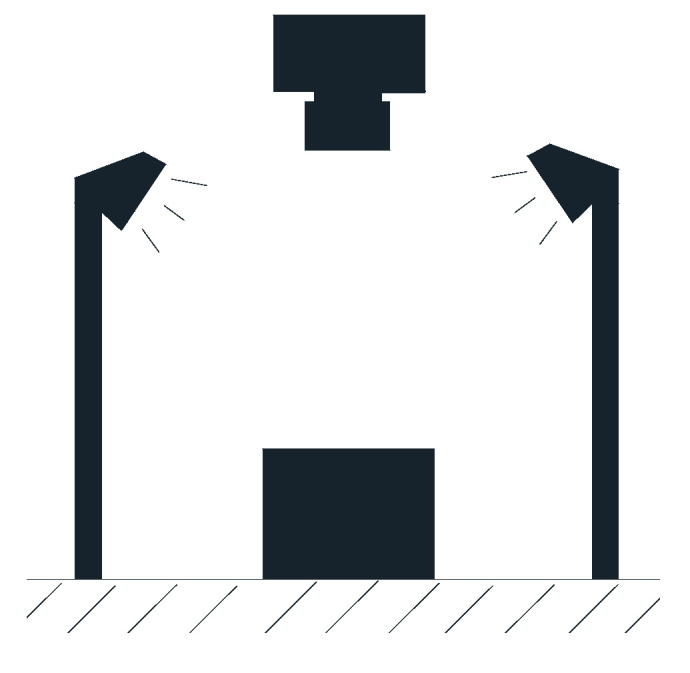
Specimen cross-section.

**Figure 8:**
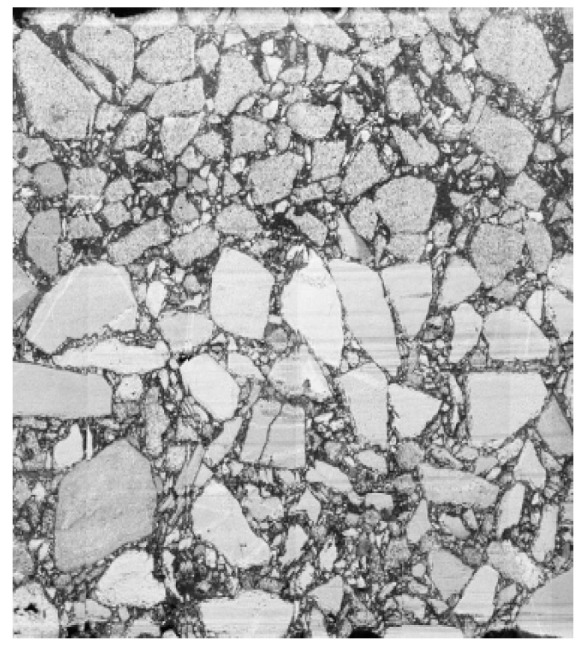
Grayscale image.

**Figure 9:**
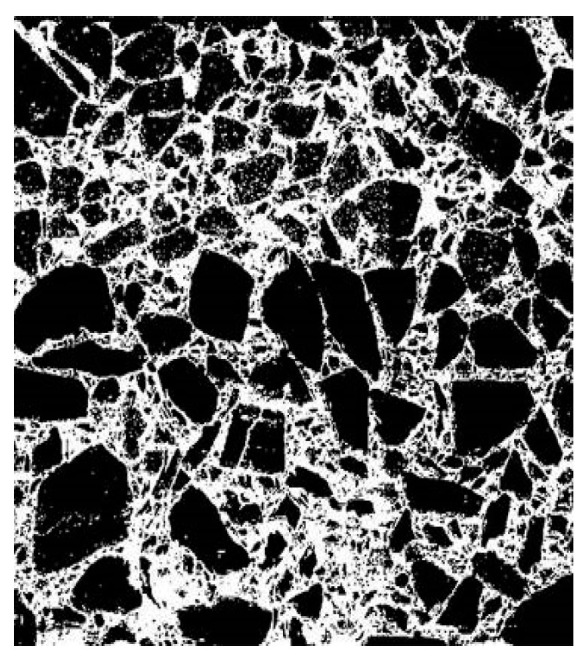
Binarized image.

In order to capture the longitudinal profile images, LED lamps were placed around the acquisition surface of the longitudinal profile to ensure the uniformity of illumination, and image acquisition was carried out by using a charge-coupled device camera, as illustrated in Figure 7. The images were then converted into grayscale images, presented in Figure 8. In the grayscale images, the gray value of each pixel ranged from 0 to 255, where 0 represents black and 255 represents white. Finally, the images of the longitudinal profiles were binarized by the adaptive threshold segmentation (Otsu) algorithm. In the binarized image, a value of 0 indicates aggregate and a value of 1 indicates asphalt mortar, as shown in Figure 9.

### 3.2. Virtual Simulation of the Uniaxial Penetration Test

**Figure 10:**
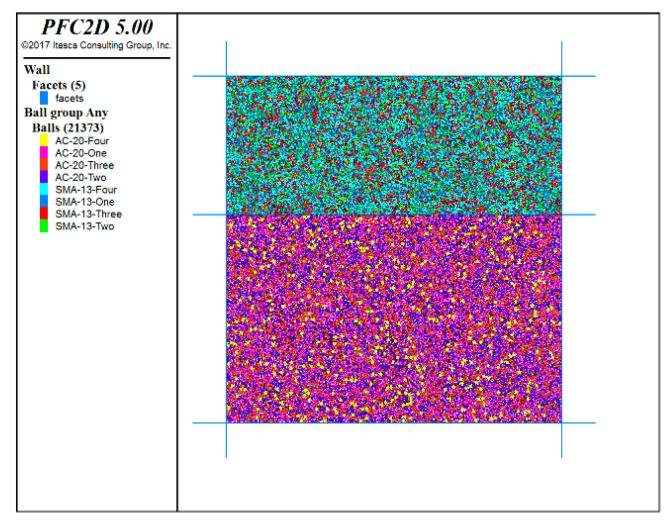
The initial model.

**Figure 11:**
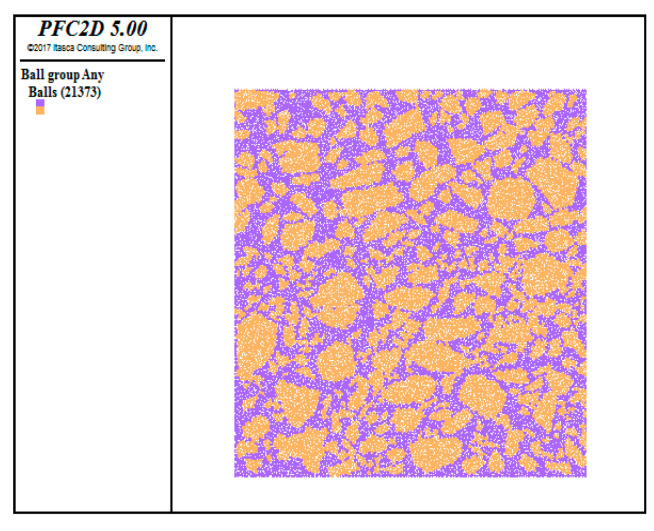
The reconfiguration model.

In this study, a friendly interactive shell (FISH) language was written to establish the initial model, including an upper layer and a lower layer, as shown in Figure 10. The pixel coordinates of the aggregates and mortar in the asphalt mixture were extracted by using MATLAB programs (R2018a, MathWorks, Natick, MA, USA). The binarized image information was then imported into the discrete element model. If the pixel value was equal to 1, the corresponding particle was asphalt mortar in the model; if the pixel value was 0, the corresponding particle was aggregate, as shown in Figure 11.

### 3.3. Determination of the Bonding Parameters

The bond model was adopted in this paper for the simulation of the asphalt mixtures. The bond between each particle in the model had adjustable tensile strengths and shear strengths to withstand different loading stresses. Bonding models consist of contact bonding models and parallel bonding models. Contact bonding means that the bond occurs only at the point of contact, and only transmits force, while the parallel bond occurs in a limited cylindrical area between the contact particles, which transmits both force and moment. In the model of this study, the contact bonding model was introduced into the asphalt mortars and the parallel bonding model was adopted between the aggregates. Based on the previous research [27], the model parameters were adjusted to the current ones based on the uniaxial penetration test. The model parameters are shown in Table 4.

## 4. Results and Discussion

### 4.1. Verification of the Virtual Uniaxial Penetration Test

**Figure 12:**
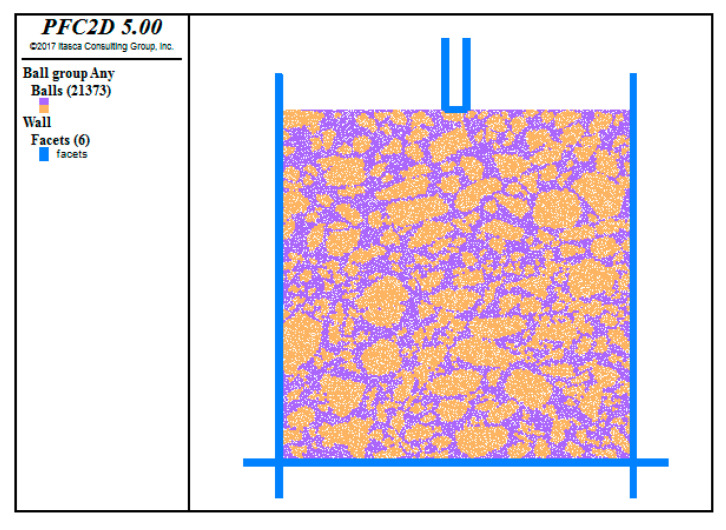
Uniaxial penetration model.

**Figure 13:**
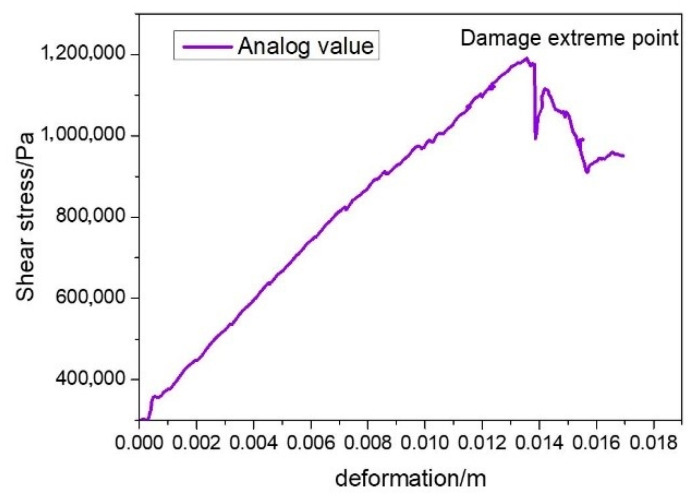
The simulation results.

The discrete element model, reconstructed by importing the binarized longitudinal profile images of the UPT specimen, was used to perform a virtual uniaxial penetration test after defining the bond parameters. Referring to the actual test, the virtual uniaxial penetration test was carried out using an indenter with a diameter of 28.5 mm, and the penetration rate of the indenter was 1 mm/min. The servo control program was written in FISH language so as to continuously adjust the speed of the structural boundary wall in order to achieve the required contact force, which was adopted as the loading way in this study. The discrete element model of the uniaxial penetration test is shown in Figure 12. The simulation results are shown in Figure 13.

To verify the virtual test results of the UPT, Table 5 shows a comparison of the laboratory and virtual test results. On average, the virtual uniaxial penetration strength of the double-layer asphalt mixture was 8.25% higher than the experimental uniaxial penetration strength, which is acceptable for the virtual simulation and for further analysis.

### 4.2. Contact Stress and Bonding Performance within the Double-Layer Asphalt Mixture

**Figure 14:**
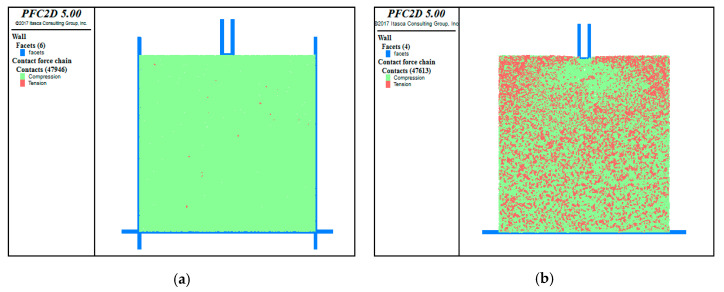
Comparison of internal contact tension and pressure distribution before and after the test. (**a**) Before the test; (**b**) After the test.

Figure 14 shows the internal contact tensile and compressive stress within the model before and after the simulation test, in which the green part indicates the contact compressive stress and the red part the contact tensile stress. After the test, a large amount of tensile stress appeared inside the model, as shown in Figure 14b. Inside the lower layer, the internal tensile and compressive stress were distributed evenly. In the upper layer, there was more compressive stress near the indenter and more tensile stress away from the indenter. The distribution of the compressive stress and tensile stress was not uniform in the upper layer. This part was more susceptible to shear deformation.

**Figure 15:**
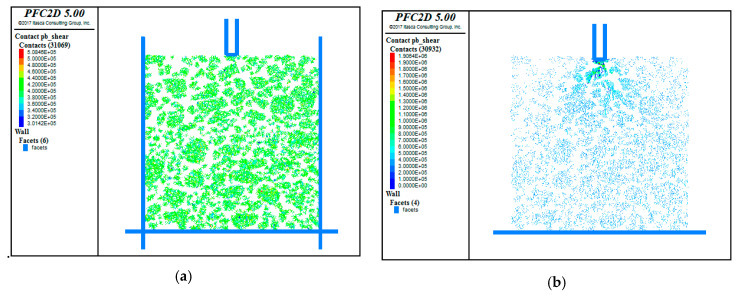
Comparison of shear stress before and after the test. (**a**) Before the test; (**b**) After the test

Figure 15 presents the change in the shear stress within the model. Before the test, the shear stress and the skeleton structure were in a relatively balanced state. Applying the virtual load introduced more shear force between the coarse aggregates in the upper layer, especially underneath the penetration head, where the shear stress increased by two to three times of that before the test. The shear stress within the lower layer was greater than that before the test, but was distributed more evenly than that in the upper layer. All of these imply that the coarse aggregates within the upper layer were subjected to greater shear stress under the load.

**Figure 16:**
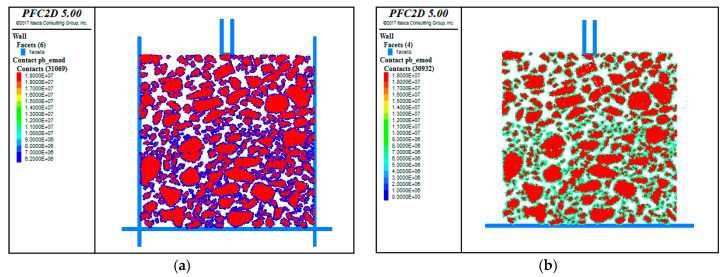
Comparison of the effective cohesive modulus of the specimens before and after the test. (**a**) Before the test; (**b**) After the test.

In this study, the viscous parameter was adopted to represent the bonding state between the coarse aggregate and the asphalt mortar. The effective bond modulus reflects the bonding ability between the aggregate and the asphalt mortar. As shown in Figure 16, the asphalt mortar wrapped around the coarse aggregates, and there was a certain bond (blue color) within the model before the test. It was evident that the penetration load made the adhesion around the coarse aggregates increase significantly (green color). The effective bond modulus after the test was larger than that before the test, but there was no significant difference in the distribution within both the upper layer and the lower layer. The bond between the coarse aggregate and the asphalt mortar was also a key factor affecting the shear resistance of the asphalt mixture.

**Figure 17:**
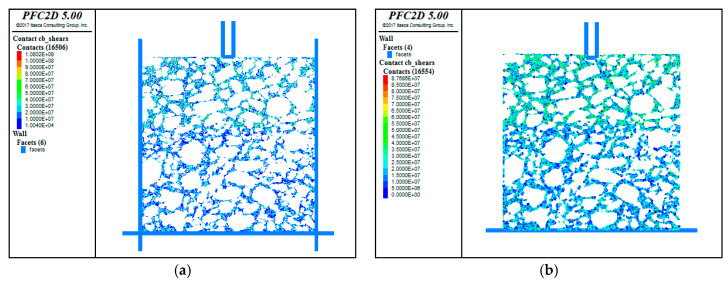
Comparison of shear stress within the asphalt mortar before and after the test. (**a**) Before the test; (**b**) After the test.

A comparison was also made on the shear stress within the asphalt mortar before and after the test in Figure 17. Even with no load applied on the model, a certain difference of the shear stress was observed within the upper layer and the lower layer. After the test, the shear stress within the upper layer was greater than that within the lower layer. It can be concluded that both the coarse aggregates and asphalt mortar contributed to shear deformation resistance, and the materials in the upper layer bore more penetration load.

### 4.3. Coarse Aggregate Movement Before and After Simulation Test

**Figure 18:**
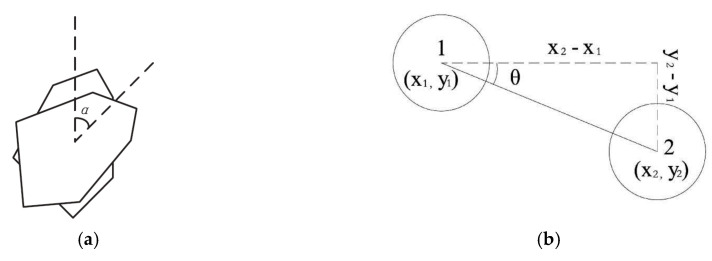
Movement of the coarse aggregate. (**a**) Rotation; (**b**) Transition.

The penetration load led to the shear deformation of the asphalt mixture, during which the coarse aggregates underwent a small range of movement, including translation and rotation, and then rearranged. To further investigate the mechanism of the shear deformation of the asphalt mixture from the perspective of the aggregate morphology, the changes in the angles and translations of the coarse aggregates were assessed by rotation angle α, vertical and horizontal translation x_2_−x_1_, y_2_−y_1_, and translation angle θ arctan ((y_2_−y_1_)/(x_2_−x_1_)), as illustrated in Figure 18, respectively. The mean displacement angle was the mean value of the displacement angles of all of the coarse aggregates in the model [28].

Table 6 shows the mean value of the rotation angle and the transition of aggregates caused by applying the penetration load. The rotation angles and transitions of the coarse aggregates in the upper layer were greater than those in the lower layer, and its macroscopic behavior was such that the shear deformation was more serious in the upper layer. The aggregate particles in the upper layer displaced vertically, while those in the lower layer generally displaced laterally under the virtual load. It directly led to a change in the aggregate skeleton and in the stability of the skeleton contributing to the shear deformation. 

This result is consistent with the previous conclusion, that shear deformation was the consequence of both densification and lateral flow deformation. Such instability of the aggregate skeleton led to the final stage of serious rutting distress.

## 5. Conclusions

In this study, a two-dimensional virtual uniaxial penetration test was built by the binarized longitudinal profile images of the UPT specimen cored from a double-layer wheel tracking test specimen to analyze the shear deformation of the asphalt mixture. The micromechanical and morphological behavior of the virtual asphalt mixture specimen during shear deformation were investigated to reveal the rutting mechanism of asphalt pavement.

Different bonding parameters were assigned to the contacts between different components within the model. The virtual test result was verified and considered to be acceptable for the virtual simulation and further analysis. Dramatic increases were observed on the tensile, compressive, shear stress, and effective bond modulus within the double-layer asphalt mixture when it was subjected to a virtual penetration load. The values of the above micromechanical parameters were greater in the upper layer, especially near the indenter, indicating that the shear deformation occurred more easily in the upper layer. The rotation and translation of the coarse aggregates within the double-layer asphalt mixture after applying the penetration load confirm that shear deformation is a process of both vertical densification deformation and lateral flow deformation. Specifically, coarse aggregates within the upper layer generate more vertical displacement, while those within the lower layer more lateral displacement.

## Figures and Tables

**Table 1:** Sieving results of the basalt aggregates and mineral powder.

Aggregate	Percentage of Mass Passing (Square Opening Screen)/%									
	16.0	13.2	9.5	4.75	2.36	1.18	0.6	0.3	0.15	0.075
1 #	100.0	91.8	22.7	0.4	0.2	0.2	0.2	0.2	0.2	0.2
2 #	100.0	100.0	99.6	20.3	3.5	2.1	1.5	0.7	0.5	0.5
3 #	100.0	100.0	100.0	90.3	10.4	4.2	1.9	1.3	1.1	0.6
4 #	100.0	100.0	100.0	99.0	81.3	63.8	41.7	24.4	13.0	5.7
Mineral powder	100.0	100.0	100.0	100.0	100.0	100.0	100.0	100.0	98.5	85.0

**Table 2:** Sieving results of the limestone aggregates and mineral powder.

Aggregate	Percentage of Mass Passing (Square Opening Screen)/%										
	26.5	19.0	13.2	9.5	4.75	2.36	1.18	0.6	0.3	0.15	0.075
1 #	100	82.6	9.2	0.6	0.6	0.6	0.6	0.6	0.6	0.6	0.6
2 #	100	100	90.5	58.2	1.7	0.6	0.3	0.3	0.3	0.3	0.3
3 #	100	100	100	100	71.7	7.9	0.3	0.3	0.3	0.3	0.3
4 #	100	100	100	100	100	77.2	60.1	35.8	17.7	16.1	10.6
Mineral powder	100	100	100	100	100	100	100	100	100	98.8	82.8

**Table 3:** Volumetric parameters and performance test results of SMA13 and AC20.

Mixture	Asphalt-Aggregate Ratio/wt.%	Stability/kN	Flow Value/(0.1 mm)	Void Ratio/%	Bulk Density/(g·cm^−3^)	Dynamic Stability/mm	Splitting Strength Ratio/%
SMA13	6.1	7.42	43.3	4.3	2.478	4631	82.7
AC20	4.6	10.49	37.87	3.6	2.454	1230	81.7

**Table 4:** The bond parameters.

Particle Stiffness/(N·m^−1^)	1 × 10^8^
Average value of contact bond strength/N	1 × 10^4^
Standard deviation of contact bond strength	0.5 × 10^4^
Average value of parallel bond strength/N	3 × 10^5^
Standard deviation of parallel bond strength	0.25 × 10^5^
Internal friction angle/°	22

**Table 5:** Comparison of virtual and laboratory test results of the uniaxial penetration strength.

Laboratory Test/MPa	Virtual Test/MPa	Error/%
1.07	1.18	10.28
1.12	1.20	7.14
1.09	1.17	7.34

**Table 6:** Mean value of the rotation angle and transition.

Movement		Upper Layer	Lower Layer
**Rotation**	Rotation angle α/°	1.99	1.37
**Transition**	Transition (x_2_−x_1_)/10^−4^ m	1.47	1.73
	Transition (y_2_−y_1_)/10^−4^ m	2.15	0.84
	Mean displacement angle θ/°	55.64	25.89
